# Immune Persistence following Primary Immunization and the Immunogenicity and Safety of a Booster Dose of a Multidose Sabin Strain-Based Inactivated Polio Vaccine in Infants Aged 18 Months

**DOI:** 10.3390/vaccines12020123

**Published:** 2024-01-25

**Authors:** Guangwei Feng, Ming Shao, Jianfeng Wang, Lili Huang, Jian Tan, Zhiwei Jiang, Wangyang You, Yurong Li, Yonghui Yang, Jing Li, Yanxia Wang

**Affiliations:** 1 Henan Provincial Center for Disease Control and Prevention, Zhengzhou 450018, China; vacfeng@163.com (G.F.); 13643826177@163.com (L.H.); dsrt12345@163.com (W.Y.); hncdcyyh2023@126.com (Y.Y.); 2 National Institute for Food and Drug Control, Beijing 100050, China; shaomingabc@163.com; 3 Sinovac Biotech Co., Ltd., Beijing 100085, China; wangjianfeng@sinovac.com (J.W.); tanj@sinovac.com (J.T.); 4 Beijing Key Tech Statistics Technology, Beijing 100025, China; zhi.wei.jiang@ktstat.com; 5 Sinovac Life Sciences Co., Ltd., Beijing 102629, China; liyr8134@sinovac.com

**Keywords:** multidose, Sabin strain, inactivated poliovirus vaccine, immune persistence, booster immunization

## Abstract

Background: The multidose Sabin-strain inactivated poliovirus vaccine (sIPV) has the potential to significantly aid in the eradication of poliomyelitis, particularly in low- and middle-income countries. As part of a phase III clinical trial in which infants were given three doses of primary immunization at 2, 3, and 4 months of age, this study aimed to evaluate immune persistence following primary immunization, as well as the safety and immunogenicity of a booster of the 5-dose sIPV in infants aged 18 months. Methods: Infants aged 18 months were given one booster dose of 5-dose sIPV in stage one, which was open-label. Unblinding was performed for stage two after completing primary immunization, which was randomized, blinded, and controlled; infants aged 18 months in the test group I–III, IPV group, and single-dose sIPV group were given one booster dose of 5-dose sIPV, conventional IPV, and single-dose sIPV, respectively, in stage two. Results: This study included 1438 infants in the immune persistence and safety set and 1387 infants in the booster per-protocol set. Fourteen months after primary immunization, the seropositivity rates (≥1:8) for types 1–3 were 100%, 99.88%, and 99.53% in the 5-dose sIPV groups; 100%, 98.97%, and 97.23% in the IPV group; and 99.66%, 100%, and 99.66% in the single-dose sIPV group. A total of 30 days after booster immunization, the seropositivity rates (≥1:8) of 3 serotypes in all the groups reached 100%. The geometric mean titers of neutralizing antibodies for types 1–3 in the 5-dose sIPV group were 9962.89, 10273, and 7870.21, with geometric mean increases of 15.76, 33.15, and 24.5, compared to the pre-booster level. The overall incidence of adverse reactions was 8.97%, with fever being the most common, observed at rates of 7.1%, 5.52%, and 7.96% in the 5-dose sIPV, IPV, and single-dose groups, respectively (*p* = 0.4845). Conclusions: The 5-dose sIPV has shown promising immune persistence and robust immune response following a booster immunization, coupled with an acceptable safety profile.

## 1. Introduction

Poliomyelitis, commonly known as polio, is an acute infectious disease caused by the poliovirus, which is classified into three serotypes: 1, 2, and 3. This disease predominantly affects humans, with children under the age of five being particularly susceptible [1]. The virus primarily targets the central nervous system, and approximately one in every 200 infections leads to irreversible paralysis. Among individuals who become paralyzed, 5–10% succumb to the disease when paralysis affects their respiratory muscles, rendering them unable to breathe [2]. At present, there is no effective cure for polio, and prevention solely relies on vaccination. Following the initiation of the Global Polio Eradication Initiative (GPEI) in 1988, the incidence of wild poliovirus has been reduced by more than 99%. This significant decrease has brought the number down from roughly 350,000 cases across over 125 endemic countries to just 14 reported cases in two endemic countries as of August 8th, 2023 [3,4]. Nonetheless, the inability to completely eradicate polio from its final strongholds poses the risk of the disease’s resurgence. A case in point is China, which, despite being declared polio-free in October 2000, experienced an outbreak of imported type 1 wild poliovirus (WPV) originating from Pakistan in the Xinjiang Uygur Autonomous Region in 2011 [5,6]. Consequently, it is imperative for China to implement strategic measures to preserve its polio-free status until the global eradication of poliomyelitis is accomplished.

Two types of polio vaccines are in use globally: the Oral Poliovirus Vaccine (OPV) and the Inactivated Poliovirus Vaccine (IPV). Both have been extensively deployed worldwide in the effort to eradicate poliovirus [5]. Although the OPV is highly effective in preventing polio, it carries certain risks due to its composition of a live attenuated vaccine virus. These risks include vaccine-associated paralytic poliomyelitis (VAPP) and, on rare occasions, vaccine-derived poliovirus (VDPV), particularly type 2 VDPV [7,8,9,10]. To simultaneously interrupt WPV transmission and reduce the risks associated with VDPVs, the World Health Organization (WHO) orchestrated a global synchronized transition from trivalent OPV (tOPV) to bivalent type 1 and type 3 OPV (bOPV) in 2016. This shift was supported by the introduction of at least one dose of an inactivated poliovirus vaccine (IPV) to address immunity gaps resulting from the withdrawal of the type 2 component [11,12,13]. Compared to OPV, the production of conventional IPV, which relies on the wild-type Salk strain, is hindered by its higher biosafety requirements and economic burden. This limitation particularly affects its coverage in low- and middle-income countries. Consequently, the WHO has recommended substituting the wild-type Salk strain with the less virulent Sabin strain, aiming to enhance the IPV’s availability and affordability [14,15].

Sinovac has developed two versions of the Sabin strain-based sIPV, cultured on Vero cells. These include a single-dose formulation (0.5 mL) and a 5-dose multidose vial formulation (2.5 mL total, 0.5 mL per shot). The single-dose sIPV by Sinovac received market approval in China on 21 July 2021. To assess the safety, immunogenicity, and lot consistency of the 5-dose sIPV, a phase III, randomized, blinded, positive-control clinical trial was conducted with infants aged two months from November 2020 to August 2021. The trial revealed that all three lots of the 5-dose sIPV exhibited consistent immunogenicity and were non-inferior in immunogenicity compared to conventional IPV and single-dose sIPV vaccines, while also demonstrating satisfactory safety profiles [16]. The results outlined in this document reflect the immune persistence following primary immunization, as well as the safety and immunogenicity of the 5-dose sIPV used for booster immunization in infants aged 18 months, as part of a phase III clinical trial.

## 2. Methods

### 2.1. Study Design

The Henan Provincial Center for Disease Control and Prevention (HNCDC) conducted this study in Xiangcheng County and Xiangfu District of Kaifeng city, Henan Province, China. The phase III study comprised two stages: the first being open-label and the second being, a randomized, blinded, positive-control trial. Considering the fact that the primary poliovirus vaccination schedule in China involves administering IPV at 2 and 3 months of age, followed by bOPV at 4 months and 4 years of age, stage one included three age groups, focusing exclusively on safety observation: adults aged 18–49 years, children aged 4 years, and infants aged 2 months (60–89 days). By contrast, the second stage was confined to just infants aged 2 months (60–89 days). In both stages, all the infants aged 2 months received three doses of the investigational vaccine at 2, 3, and 4 months for primary immunization and a booster dose at 18 months. The booster immunization, conducted from March to October 2022, was open-label. This study aimed to evaluate the immune persistence after primary immunization and to assess the safety and immunogenicity of the investigational vaccine after booster immunization. The clinical trial protocol and the informed consent form were reviewed and approved by the Ethics Committee of the HNCDC (2019-YM-012-02). Additionally, the clinical trial was registered at ClinicalTrials.gov (accessed on 17 January 2024) (NCT05386810). For more details on the study design, please refer to the Supplementary Material.

### 2.2. Participants

Participants aged 18 months from both stages, having completed the full primary immunization schedule, were summoned back to the Xiangcheng County CDC and Xiangfu District CDC for booster immunization. Before the booster immunization, the main exclusion criteria for participants included the following: (1) receiving any polio vaccine following primary immunization completion; (2) experiencing any serious adverse events (SAEs) related to the investigational vaccine; (3) suffering severe allergic reactions post-vaccination; and (4) other conditions deemed by researchers as rendering participants unsuitable for participation. The legal guardians of each infant provided signed informed consent before their enrollment.

### 2.3. Randomization and Blinding

Stage one was open-label throughout the study, and participants in stage two during primary immunization were randomly assigned to test group I (Lot 1 no. 201907001), test group II (Lot 2 no. 201907002), test group III (Lot 3 no. 201907003), IPV control group (Lot no. R3N141M[R3N14]), and single-dose sIPV control group (Lot no. 2019004) at a ratio of 1:1:1:1:1. During primary immunization, the participants received the investigational vaccine of different lots or control vaccines in a blinded condition. Following the completion of a 30-day safety observation period after the primary immunization, the measurements of neutralizing antibodies, data cleaning, database locking were conducted, and unblinding was performed on 4 January 2022.

The booster immunization phase was open-label, with no randomization involved. Differing from primary immunization, participants in test group I–III received the same lot of the investigational vaccine (Lot no. F202112001), while those in the IPV control group and the single-dose sIPV control group were administered IPV (Lot no. U3E331M) and single-dose sIPV (Lot no. 202111004), respectively.

### 2.4. Vaccines

The investigational 5-dose vial sIPV, developed by Sinovac Biotech Company, is an inactivated trivalent vaccine. It is produced as a liquid formulation, each dose comprising 15, 45, and 45 D antigen units (DU) for types 1, 2, and 3 Sabin polioviruses, respectively, all cultured on Vero cells (2.5 mL for five doses, 0.5 mL/dose). To mitigate the risk of contamination, especially since a single vial may be used multiple times, the vaccine includes the preservative 2-phenoxyethanol. The control conventional IPV, produced in a single-dose form (0.5 mL/dose) by Sanofi Pasteur, utilizes types 1 (Mahoney strain), 2 (MEF-1 strain), and 3 (Saukett strain) polioviruses, also cultured on Vero cells. The antigen contents for these strains are 40, 8, and 32 DU, respectively. Additionally, Sinovac’s single-dose sIPV (0.5 mL/dose) is manufactured using the same process as the investigational 5-dose vial sIPV but without the inclusion of the preservative 2-phenoxyethanol. For all three vaccines, the booster injection was administered intramuscularly.

### 2.5. Procedures

Before booster immunization, the investigators verified whether the recalled participants had received any other polio vaccines through the Expanded Programme on Immunization (EPI) system. They also consulted the clinical trial management system (CTMS) to review the prior investigational data of the participants, determining their eligibility for the trial.

In stage one, 18-month-old infants were administered a single booster dose of the investigational vaccine. For each participant, only one dose was used from a 5-dose vial, and any leftover vaccine was discarded. In stage two, infants aged 18 months in test groups I–III, along with those in the IPV control and single-dose sIPV groups, received a single booster dose of the corresponding vaccine.

Immediate adverse events (AEs) were monitored on-site for 30 min after vaccination for all the participants. Systemic and solicited local AEs within 0–7 days, unsolicited AEs within 0–30 days post-vaccination, and serious AEs (SAEs) up to 30 days after booster vaccination were all documented.

AEs were identified through both active follow-up and passive monitoring, with participants recording events on contact cards. Three follow-up visits were conducted within the first 7 days post-vaccination—two via telephone at 6–24 h and 4–7 days, and one face-to-face within 1–3 days—followed by weekly telephone visits within 8–30 days.

Solicited local AEs included tenderness, redness, nodules, erythema, swelling, rashes (injection site), and induration and pruritus, while solicited systemic AEs encompassed fever (axillary temperature), acute allergic reactions, skin and mucosal abnormalities, irritability or suppression, new-onset seizures, anorexia, and vomiting.

In stage one, no blood samples were collected. In stage two, approximately 2.5 mL of blood was drawn from each participant both before (day 0) and 30 days after (day 30) the booster vaccination. To evaluate the immunogenicity of the vaccines, titers of types 1, 2, and 3 poliovirus-specific neutralizing antibodies were measured using a microneutralization assay. Seroconversion was defined as either a change from seronegative (<1:8) to seropositive (≥1:8) status, or a four-fold increase from baseline titers if already seropositive. Additionally, seropositivity rates (≥1:8) were reported.

### 2.6. Laboratory Testing

The neutralization assay was carried out by the National Institutes for Food and Drug Control. The process involved mixing serial dilutions of inactivated serum with 100 50% cell culture infective doses (CCID50) of the poliovirus (Sabin strain, types I, II, or III) in 96-well plates. This mixture was incubated at 35.0 °C 5% CO_2_ incubator for 3 h, followed by the addition of a Hep-2 cell suspension (control the cell concentration at 0.8~1.0 × 10^5^ cells/mL). The plates were then incubated for 5 to 7 days, after which the neutralizing antibody titer was identified. The titer was defined as the highest dilution of the serum that protected 50% of the culture against 100 CCID50 of the poliovirus.

### 2.7. Outcomes

The trial had two main endpoints: immunogenicity and safety. The immunogenicity endpoints encompassed seropositivity rates (SPRs) at ≥1:8, geometric mean titers (GMTs) of neutralizing antibodies before booster immunization (14 months after primary immunization) and 30 days after booster immunization, as well as seroconversion rates (SCRs) and geometric mean increases (GMIs) 30 days after booster immunization. The safety endpoints included the incidence of adverse reactions (ARs) within 0–7 days and 0–30 days after vaccination, as well as the incidence of serious adverse events (SAEs) during the safety observation period, which extended from 30 days after primary immunization to 30 days after booster immunization.

### 2.8. Statistical Analysis

The methodology for calculating the sample size was outlined in a previous publication [16]. The statistical analyses were performed using the SAS 9.4 Software (SAS Institute Inc., Cary, NC, USA). A two-sided 95% confidence interval was calculated using the Clopper–Pearson method. The Chi-square test/Fisher’s exact probability test was used to statistically compare the differences in SPRs (≥1:8) and SCRs among groups. An analysis of variance after logarithmic transformation was used to statistically compare the differences in GMT and GMI among groups. Adverse events were encoded using MedDRA version 24.1, and the Fisher’s exact probability test was used to statistically compare the incidence of ARs among groups. Test groups 1–3 were combined and were referred to as the 5-dose sIPV group during analysis. A *p*-value of less than 0.05 was considered statistically significant.

## 3. Results

### 3.1. Study Population

In stage one, 72 participants were enrolled in the study, with 24 participants in each of the three age groups. Only the infant group received booster immunization, and all 24 participants received the vaccination at 18 months of age and were included in the safety set without any dropouts.

**Figure 1:**
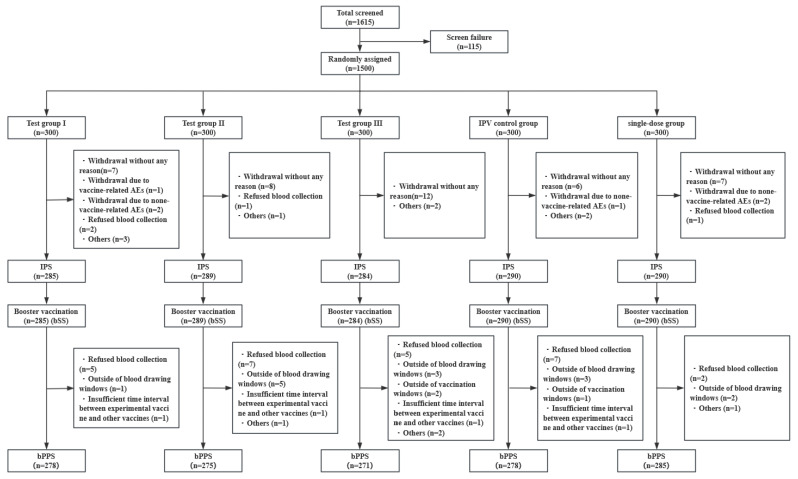
Flow chart of the participants in stage two included in the IPS, bSS, and bPPS.

In stage two, a total of 1500 participants were enrolled in this study. Of these, 1438 (95.87%) completed primary immunization and blood collection before booster immunization and were included in the full analysis set for the booster (bFAS) and the immune persistence set (IPS). Additionally, 1438 (95.87%) participants completed the full-schedule immunization (3 doses of the primary immunization and 1 dose of the booster immunization) without dropping out and were included in the safety analysis set for the booster (bSS). Furthermore, 1387 (92.47%) participants completed the full-schedule immunization and two blood collections (before booster vaccination and 30 days after booster vaccination) without violating the trial protocol and were included in the booster per-protocol set (bPPS). The flow chart of the participants is shown in Figure 1. At enrollment, the mean age was 2.41 months in the 5-dose sIPV group, 2.39 months in the conventional IPV group, and 2.4 months in the single-dose sIPV group. Further information about participant characteristics is provided in Table 1.

### 3.2. Immune Persistence

Fourteen months after primary immunization (before booster immunization), the SPRs (≥1:8) in the 5-dose sIPV group, the conventional IPV group, and the single-dose sIPV were 100%, 100%, and 99.66% for type 1; 99.88%, 98.97%, and 100% for type 2; and 99.53%, 97.23%, and 99.66% for type 3. The SPRs (≥1:8) for type 3 in the 5-dose sIPV group and the single-dose group were higher than that in the conventional IPV group, and the differences were statistically significant (*p* = 0.0298). The GMTs for types 1, 2, and 3 were 642.57, 312.63, and 325.81 (GMI 0.23, 0.67, and 0.16) in the 5-dose test group; 193.05, 142.34, and 130.54 (GMI 0.35, 0.73, and 0.13) in the conventional IPV control group; and 701.65, 327.51, and 365.23 (GMI 0.23, 0.63, and 0.16) in the single-dose sIPV control group, showing a decreasing trend over time and being lower than those at 30 days after primary immunization. Additional details are provided in Table 2.

### 3.3. Booster Immunogenicity

A total of 30 days after booster immunization, the SPRs (≥1:8) of all 3 serotypes reached 100% in all the groups, and the SCRs for types 1, 2, and 3 were 91.26%, 92.45%, and 89.47% in the 5-dose sIPV group, the conventional IPV group, and the single-dose sIPV group, respectively, for type 1; 97.82%, 94.24%, and 98.25% for type 2; and 93.81%, 96.40%, and 92.28% for type 3. The SCR for type 2 was higher in the 5-dose sIPV group than in the conventional IPV group, and the difference was statistically significant (*p* = 0.0029). The GMTs for types 1–3 were 9962.89, 10273, and 7870.21 (GMI 15.76, 33.15, and 24.5) in the 5-dose sIPV group; 4086.46, 4141.8, and 6844.97 (GMI 20.73, 29.03, and 51.32) in the conventional IPV control group; and 10202.27, 10859.77, and 8046.47 (GMI 14.59, 33.19, and 22.24) in the single-dose sIPV control group, indicating strong immune responses that were also much higher than those observed 30 days after primary immunization. More details are provided in Table 3.

### 3.4. Safety

In stage one, the overall incidences of ARs within 7 days following booster immunization was 4.17% (1/24) in the infant group. In stage two, AEs occurred in 16.67% (143/858), 15.17% (44/290), and 17.24% (50/290) of participants after booster immunization in the 5-dose sIPV group, conventional IPV group, and single-dose sIPV, respectively. The incidences of ARs were 8.97% (77/858), 7.24% (21/290), and 9.31% (27/290) in the 5-dose sIPV group, conventional IPV group, and single-dose sIPV, respectively, and the difference was not statistically significant (*p* = 0.6237). ARs after booster immunization occurred mainly within 0–7 days and were mainly solicited. The most common AR was fever; the incidences of fever were 7.10% (61/858), 5.52% (16/290), and 7.96% (23/290) in the 5-dose sIPV group, conventional IPV group, and single-dose group, respectively, and the difference was not statistically significant (*p* = 0.4845). The overall incidence of other ARs, including diarrhea (0.76%) and vomiting (0.56%), was considerably lower than that of fever, with no significant differences noted across the groups. The ARs were mainly grade I and II in severity, and the overall incidences of grade I, II, and III ARs were 3.89% (56/1438), 5.01% (72/1438), and 0.07% (1/1438), respectively, and the only grade III AR was fever. Further information is provided in Table 4. SAEs were reported during the safety observation period. Between 14 months after primary immunization (before booster immunization) and 30 days after booster immunization in stage one, only one SAE was reported, which was diagnosed as mycoplasmal pneumonia. In stage two, the incidences of SAEs in the 5-dose sIPV group, conventional IPV group, and single-dose sIPV group were 0.58% (5/858), 1.03% (3/290), and 0.35% (1/290), respectively, with no significant difference noted across the groups (*p* = 0.638). The majority of the SAEs reported in stage two were cases of pneumonia. According to the investigators’ assessments, none of the reported SAEs were related to vaccination.

Between 30 days and 14 months after primary immunization in stage one, only two SAEs occurred in the infant group. During stage two, the incidences of SAEs were 10.58% (95/898), 10% (30/300), and 13.76% (41/298) in the 5-dose sIPV group, conventional IPV group, and single-dose sIPV group, respectively, with no statistically significant difference among the groups (*p* = 0.2609). No vaccine-related SAEs occurred, and the majority of the SAEs were pneumonia.

## 4. Discussion

The WHO advocates for the inclusion of at least two doses of IPV in the vaccination schedules of countries employing OPV in their national immunization programs. This recommendation aims to diminish the risks associated with VAPP and to establish a foundational immunity that can be quickly enhanced in the event of a poliovirus type 2 outbreak, particularly following the removal of the type 2 virus component from OPV [17,18,19]. The inclusion of IPV is especially critical for middle- and low-income countries, including Pakistan and Afghanistan, which not only border China but also continue to be polio-endemic regions. In these countries, the 5-dose sIPV presents a more accessible and cost-effective alternative compared to both conventional IPV and single-dose sIPV. Consequently, this renders the 5-dose sIPV a more viable option for inclusion in their vaccination programs [20,21].

This study’s findings suggest a robust immune persistence with the 5-dose sIPV. Fourteen months after primary immunization (before the booster), GMTs for all the serotypes in the 5-dose sIPV group had decreased by 16% to 67% compared to their levels 30 days after primary immunization. However, the SPRs for all the serotypes in this group were nearly 100% (more than 99.53%), which were comparable to the rates observed 30 days after primary immunization (up to 100%). Additionally, the SPRs for type 3 in both the 5-dose and single-dose sIPV groups were statistically higher than those in the conventional IPV group, suggesting that sIPV may provide enhanced long-term protection against type 3 virus, post-primary immunization. In a separate study assessing IPV’s immune persistence in China, the SPRs for serotypes 1–3 in the conventional IPV group were 98.3%, 94.7%, and 94.9% at 18–24 months, marginally lower than our findings [22]. Another study on the immune persistence of DTaP–IPV//PRP∼T at 18–20 months reported SPRs for types 1–3 Salk-strain poliovirus antibodies at 94%, 88.4%, and 87.6%, which were also lower than those observed in this study [23].

This study demonstrated a robust immune response following the booster dose of the 5-dose sIPV because, 30 days after booster immunization, the SPRs for all three serotypes in all the groups reached 100%. Notably, the SCR of type 2 poliovirus in the 5-dose sIPV group was statistically higher compared to the conventional IPV group, suggesting that sIPV elicits a stronger immune response against type 2 virus after the booster dose, thereby providing enhanced protection. The GMTs of neutralizing antibodies for serotypes 1–3 were 9962.89, 10,273, and 7870.21, respectively, representing an increase of 15.76 to 33.15 times the levels observed before booster immunization. This increase significantly exceeded the GMTs recorded 30 days after primary immunization (for serotypes 1–3: 2716.97, 459.71, 1998.23). In a comparable study, 30 days following booster vaccination, all the children in the IPV group exhibited seroprotective antibodies for each poliovirus, with GMTs increasing by at least 22-fold for each type [22]. In a 2012–2014 phase III clinical trial of sIPV in China, the antigen contents for types I, II, and III were established at 30, 32, and 45 D-antigen units (DU), respectively. The seroconversion rates (SCRs) for serotypes 1–3 in the test group reached 94.2%, 92.4%, and 93.0%. By contrast, with the 5-dose sIPV containing 15, 45, and 45 D antigen units for the respective serotypes, the SCRs were 91.26%, 97.82%, and 93.81%, respectively, 30 days post-booster dose. This indicates a positive correlation between the level of immune responses and antigen content [24].

In this study, drawing from stage two findings, the 5-dose sIPV demonstrated safety profiles comparable to both the conventional IPV group and the single-dose sIPV in terms of booster dose safety, with no SAEs related to the vaccination. The overall incidence of ARs after booster immunization within 0–30 days was 8.97% in the 5-dose sIPV group, compared to 12.17%, 12.80%, and 12.63% after the first to third doses of primary immunization, indicating improved safety profiles following the booster vaccination. This improvement may be attributed to the participants’ age at the time of vaccination. Most of the ARs were mild to moderate in severity, with only one grade III AR observed in the 5-dose sIPV group. The most common AR was fever, though the difference was not statistically significant. In a previous phase III trial of sIPV in China, the overall incidence of AEs after booster vaccination at 18 months was 37.2%, significantly higher than in the Salk-IPV control group and markedly higher than in our study (16.67%). This discrepancy is likely due to differences in antigen content and composition [24].

This study encompasses several limitations. First, it exclusively employed the Sabin strains, the basis for the sIPV, in detecting neutralizing antibodies induced by the three vaccines. Consequently, this approach may have led to the underestimation of GMTs in conventional IPV, which contains Salk strains. However, earlier research on Sinovac’s single-dose sIPV has shown a satisfactory cross-neutralization capacity against various WPV strains [25]. Secondly, long-term follow-up studies to assess the immune persistence of the 5-dose sIPV have not been conducted. However, our previous phase IV study on single-dose sIPV in 4-year-old children showed that Serum Protection Rates (SPRs) for neutralizing antibodies against serotypes I–III reached 100.00% in all participants [26]. Similarly, another recent study reported that sIPV sustained a 100.00% SPR for neutralizing antibodies against these serotypes for at least 10 years post-booster immunization [27]. Future research will focus on the extended immune persistence of the 5-dose sIPV. Thirdly, the open-label nature of the booster phase could introduce a potential bias. Nonetheless, despite the unblinding, the participants and investigators involved in the follow-up, relevance determination, and laboratory testing remained blinded, except for principal investigators, statisticians, and sponsors, to ensure the maximum preservation of the blind state as far as possible. Finally, it is crucial to recognize that countries endemic for WPV and those at a high risk of WPV importation are predominantly low-income and underdeveloped regions. These areas are in the greatest need of the multi-dose sIPV. The WHO advises against these regions switching to an IPV-only schedule, in order to reduce the risk of undetected transmission. Consequently, further investigation into sequential immunization using the 5-dose sIPV, particularly its co-administration with routine immunization vaccines, is essential [5].

## 5. Conclusions

The 5-dose vial sIPV demonstrated significant immune persistence fourteen months after primary immunization and effectively elicited robust immune responses against all three poliovirus serotypes after booster immunization. Additionally, the safety profile of the booster immunization was satisfactory, aligning with that of the control groups. Licensing this 5-dose vial sIPV holds the potential to substantially contribute to the worldwide endeavor to eradicate polio.

## Figures and Tables

**Table 1:** Participant demographics and other baseline information.

Characteristics (Stage One) (bSS)	Infants Aged 2 Months (n = 24)			
Age at enrollment (days), mean (SD)	2.47 (0.26)			
Male n (%)	10 (41.67)			
Axillary temperature (°C), mean (SD)	36.65 (0.24)			
Height (cm), mean (SD)	61.02 (2.23)			
Weight (kg), (mean (SD)	6.52 (0.68)			
**Characteristics (Stage two) (bSS)**	**5-dose sIPV (n = 858)**	**IPV (n = 290)**	**Sing-dose sIPV (n = 290)**	***p* Value**
Age at enrollment (days), mean ± SD)	2.41 (0.27)	2.39 (0.26)	2.41 (0.25)	0.5502
Han ethnic n (%)	854 (99.53)	289 (99.66)	290 (100.00)	0.8266
Male n (%)	455 (53.03)	141 (48.62)	159 (54.83)	0.2898
Axillary temperature (°C), mean (SD)	36.66 (0.24)	36.66 (0.23)	36.66 (0.23)	0.9925
Height (cm), mean (SD)	60.52 (2.34)	60.31 (2.49)	60.64 (2.56)	0.2433
Weight (kg), mean (SD)	6.36 (0.79)	6.30 (0.83)	6.41 (0.79)	0.2278

**Table 2:** SPRs (≥1:8), GMTs, and GMIs in the immune persistence set fourteen months after primary immunization (IPS).

Variable	5-Dose sIPV (n = 858)	IPV (n = 290)	Sing-Dose sIPV (n = 290)	*p* Value
Serotype 1				
SPRs (≥1:8) (95%CI)	100.00 (99.57, 100.00)	100.00 (98.74, 100.00)	99.66 (98.09, 99.99)	0.4033
GMTs (95%CI)	642.57 (593.51, 695.69)	193.05 (169.94, 219.30)	701.65 (611.75, 804.77)	<0.0001
GMIs (95%CI)	0.23 (0.22, 0.25)	0.35 (0.31, 0.40)	0.23 (0.20, 0.26)	<0.0001
Serotype 2				
SPRs (≥1:8) (95%CI)	99.88 (99.35, 100.00)	98.97 (97.01, 99.79)	100.00 (98.74, 100.00)	0.0652
GMTs (95%CI)	312.63 (290.67, 336.25)	142.34 (124.43, 162.83)	327.51 (287.52, 373.07)	<0.0001
GMIs (95%CI)	0.67 (0.63, 0.72)	0.73 (0.64, 0.84)	0.63 (0.56, 0.72)	0.2454
Serotype 3				
SPRs (≥1:8) (95%CI)	99.53 (98.81, 99.87)	97.23 (95.55, 99.24)	99.66 (98.09, 99.99)	0.0298
GMTs (95%CI)	325.81 (300.15, 353.67)	130.54 (112.45, 151.53)	365.23 (320.12, 416.69)	<0.0001
GMIs (95%CI)	0.16 (0.15, 0.18)	0.13 (0.11, 0.15)	0.16 (0.14, 0.19)	0.0076

**Table 3:** SPRs (≥1:8), SCRs, GMTs, and GMIs in the booster per-protocol set for 30 days after booster immunization (bPPS).

Variable	5-Dose sIPV (n = 824)	IPV (n = 278)	Sing-Dose sIPV (n = 285)	*p* Value ^a^	*p* Value ^b^
Serotype 1					
SPRs (≥1:8) (95%CI)	100.00 (99.55, 100.00)	100.00 (98.68, 100.00)	100.00 (98.71, 100.00)	/	/
SCRs (95%CI)	91.26 (89.12, 93.10)	92.45 (88.68, 95.26)	89.47 (85.31, 92.78)	0.5389	0.3661
GMTs (95%CI)	9962.89 (9530.88, 10,414.49)	4086.46 (3686.81, 4529.43)	10202.27 (9509.51, 10,945.49)	<0.0001	0.5877
GMIs (95%CI)	15.76 (14.54, 17.09)	20.73 (17.76, 24.20)	14.59 (12.78, 16.67)	0.0012	0.3378
Serotype 2					
SPRs (≥1:8) (95%CI)	100.00 (99.55, 100.00)	100.00 (98.68, 100.00)	100.00 (98.71, 100.00)	/	/
SCRs (95%CI)	97.82 (96.57, 98.70)	94.24 (90.82, 96.67)	98.25 (95.95, 99.43)	0.0029	0.6720
GMTs (95%CI)	10273.00 (9883.32, 10,678.04)	4141.80 (3728.04, 4601.49)	10859.77 (10,230.53, 11,527.72)	<0.0001	0.1436
GMIs (95%CI)	33.15 (30.68, 35.82)	29.03 (24.87, 33.89)	33.19 (29.13, 37.82)	0.1050	0.9884
Serotype 3					
SPRs (≥1:8) (95%CI)	100.00 (99.55, 100.00)	100.00 (98.68, 100.00)	100.00 (98.71, 100.00)	/	/
SCRs (95%CI)	93.81 (91.94, 95.36)	96.40 (93.48, 98.26)	92.28 (88.55, 95.10)	0.1025	0.3708
GMTs (95%CI)	7870.21 (7507.80, 8250.10)	6844.97 (6253.64, 7492.22)	8046.47 (7455.01, 8684.86)	0.0046	0.6359
GMIs (95%CI)	24.50 (22.44, 26.75)	51.32 (43.96, 59.92)	22.24 (19.28, 25.64)	<0.0001	0.2655

**^a^** The *p* values were calculated for the comparison of the 5-dose sIPV group and IPV group. **^b^** The *p* values were calculated for the comparison of the 5-dose sIPV group and single-dose sIPV group.

**Table 4:** Overall profiles of ARs within 30 days after booster immunization in the safety set (bSS).

**Stage one**	**Infants aged 18 months (n = 24)**			
Overall	1 (4.17)			
Solicited	1 (4.17)			
Local	1 (4.17)			
Systemic	0 (0.00)			
Unsolicited	0 (0.00)			
**Stage two**	**5-dose sIPV** **(n = 858)**	**IPV** **(n = 290)**	**Sing-dose sIPV** **(n = 290)**	***p*** **Value**
Overall	77 (8.97)	21 (7.24)	27 (9.31)	0.6237
Solicited	72 (8.39)	20 (6.90)	24 (8.28)	0.7369
Systemic	66 (7.69)	19 (6.55)	24 (8.28)	0.7390
Fever	61 (7.10)	16 (5.52)	23 (7.96)	0.4845
Vomiting	3 (0.35)	2 (0.69)	3 (1.04)	0.2519
Irritability	1 (0.12)	1 (0.34)	1 (0.34)	0.3567
Acute allergic reaction	1 (0.12)	0 (0.00)	0 (0.00)	1.0000
Local	6 (0.70)	1 (0.34)	0 (0.00)	0.4480
Redness	5 (0.58)	1 (0.34)	0 (0.00)	0.6352
Tenderness	1 (0.12)	0 (0.00)	0 (0.00)	1.0000
Unsolicited	5 (0.58)	1 (0.34)	5 (1.73)	0.1375
Diarrhea	5 (0.58)	1 (0.34)	5 (1.73)	0.1230

## Data Availability

Data are contained within the article.

## Supplementary Materials

For more details, please see the full clinical trial protocol: http://www.sinovac.com/en-us/news/id-3221 (accessed on 18 January 2024).
